# Biophysical modeling identifies an optimal hybrid amoeboid-mesenchymal mechanism for maximal T cell migration speeds

**DOI:** 10.1016/j.celrep.2025.116824

**Published:** 2026-01-12

**Authors:** Roberto Alonso-Matilla, Diego I. Pedro, Alfonso Pepe, Jose Serrano-Velez, Michael Dunne, Duy T. Nguyen, W. Gregory Sawyer, Paolo P. Provenzano, David J. Odde

**Affiliations:** 1 Department of Biomedical Engineering, University of Minnesota, Minneapolis, MN, USA; 2 University of Minnesota Physical Sciences in Oncology Center, Minneapolis, MN, USA; 3 University of Minnesota Center for Multiparametric Imaging of Tumor Immune Microenvironments, Minneapolis, MN, USA; 4 Masonic Cancer Center, University of Minnesota, Minneapolis, MN, USA; 5 Department of Hematology, Oncology, and Transplantation, University of Minnesota, Minneapolis, MN, USA; 6 Stem Cell Institute, University of Minnesota, Minneapolis, MN, USA; 7 Department of Bioengineering, H. Lee Moffitt Cancer Center and Research Institute, Tampa, FL, USA; 8 Department of Medical Engineering, University of South Florida, Tampa, FL, USA; 9 Lead contact

## Abstract

Despite recent advances in cell migration mechanics, the principles governing rapid T cell movement remain unclear. Efficient migration is critical for antitumoral T cells to locate and eliminate cancer cells. To investigate the upper limits of cell speed, we develop a hybrid stochastic-mean field model of bleb-based cell motility. Our model suggests that cell-matrix adhesion-free bleb migration is highly inefficient, challenging the feasibility of adhesion-independent migration as a primary fast mode. Instead, we show that T cells can achieve rapid migration by combining bleb formation with adhesion-based forces. Supporting our predictions, three-dimensional gel experiments confirm that T cells migrate significantly faster under adherent conditions than in adhesion-free environments. These findings highlight the mechanical constraints of T cell motility and suggest that controlled modulation of tissue adhesion could enhance immune cell infiltration into tumors. Our work provides insights into optimizing T cell-based immunotherapies and underscores that indiscriminate antifibrotic treatments may hinder infiltration.

## In brief

In this work, Alonso-Matilla et al. present a model of T cell motility showing that combining blebbing with moderate adhesion enables fast migration in complex microenvironments. Experiments in collagen-coated microgels validate that adhesion enhances speed, revealing mechanical limits of T cell motility and opportunities to improve immune infiltration into tumors.

## Graphical abstract

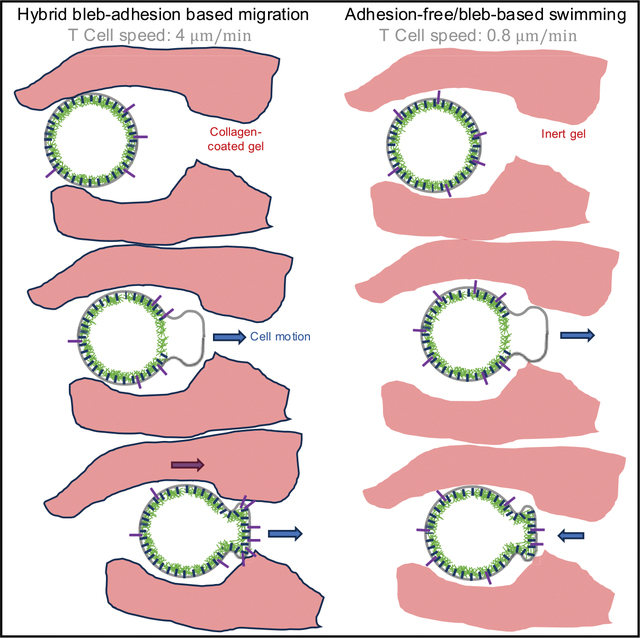

## INTRODUCTION

Cell migration is a complex multistep process that is critical for *in vivo* processes such as morphogenesis,^1^ maintaining tissue health and homeostasis,^2^ and driving diseases such as cancer, including cancer cell dissemination during metastasis^3^ and immune cell migration within tumors.^4^ Single cells migrating in three-dimensional (3D) environments use two dominant modes of migration: amoeboid and mesenchymal.^5^ The amoeboid phenotype is characterized by a rounded cellular morphology, high cortical contractility, low cell-matrix adhesions, and narrow/tight extracellular spaces, and it is commonly associated with the formation of blebs,^6–13^ which are pressure-driven hemi-spherical cellular membrane protrusions that are initiated by either a local membrane-cortex detachment or a local cortex rupture.^14–16^ This amoeboid blebby phenotype has been reported under some conditions for transformed cells (e.g., melanoma and breast and squamous carcinoma cells *in vivo*^17–20^) and is frequently utilized by T cells.^21–23^ The mesenchymal-like phenotype is frequently displayed by fibroblasts and multiple cancer types, including carcinomas, fibrosarcomas, and glioblastomas.^24,25^ It is characterized by an elongated morphology, strong cell-matrix adhesion, and the formation of actin-rich leading-edge structures such as spike-like filopodia or broad flattened lamellipodia.^10,12,24,26^ Cells navigate through extremely complex microenvironments, such as tight porous viscoelastic tissues or narrow vascular pores. As a result, many cancer cells^5,12,17,27–31^ and immune cells, including T cells,^21^ can extend both types of cellular protrusions and adapt to different mechanical and chemical microenvironments by transitioning between mesenchymal and amoeboid migration phenotypes.

T cell-based cancer therapies offer a promising approach in the fight against cancer.^32^ However, successful T cell-based therapies have been limited mainly to hematologic cancers. Solid tumors create a highly immunosuppressive and fibrotic microenvironment that hinders the physical infiltration and activity of cytotoxic T cells, thus enabling tumor growth and metastasis.^33,34^ Despite the significant progress in understanding bleb formation in recent years,^21,35,36^ it remains largely unknown how bleb-producing T cells exchange momentum with their surroundings to navigate through challenging mechanical and chemical microenvironments such as tumors.

Adhesion-dependent migration, described and mathematically modeled using a motor-clutch framework,^25^ has long been thought to be required for cell motility,^37^ but some recent findings have challenged this view.^11,26,38–51^ Following the seminal paper by Lämmermann et al.,^38^ which demonstrated that leukocyte migration *in vitro* and *in vivo* is independent of various integrin heterodimers, there has been an ongoing debate about whether the rapid migration of immune cells, particularly those exhibiting an amoeboid phenotype, requires adhesion-based forces.^40,44,48,51^ The question of whether immune cells can swim through tissues autonomously or if they depend on other adhesion molecules besides integrins (e.g., CD44) for migration remains unresolved. Notably, T cells are capable of achieving exceptionally high migration speeds, with *in vivo* motility coefficients of approximately 10 *μ*m^2^/min,^21^ a phenomenon that remains poorly understood and raises questions about potential limits imposed on standard adhesion-based motor-clutch migration mechanisms by diffusion-limited F-actin polymerization rates. Additionally, while adhesion-independent models have been proposed, a recent study^52^ has shown that T cells can generate measurable contractile forces on compliant substrates, suggesting that traction forces and substrate interactions may still play an important role during T cell migration.

Most animal cells have a dense, thin actomyosin layer underneath the plasma membrane that produces cortical tension-mediated forces that are transmitted to the membrane by membrane-cortex linker proteins, such as Ezrin, Radixin, and Moesin.^53,54^ Upon local membrane-cortex cohesion loss, cortical forces no longer get transmitted to the plasma membrane, and a bleb nucleates and undergoes rapid expansion, reaching bleb sizes on the order of ~1 μm. At the bleb nucleation site, the local hydrostatic pressure presumably drops, and cytoplasmic material flows from the center of the cell to the low-pressure bleb site.^55,56^ The bleb cycle ends by the self-assembly of new cortex underneath the bleb membrane, which drives bleb retraction.^14,57^ Yet, it is not obvious whether this form of cell motility requires cell-matrix adhesion interactions or non-specific cell-matrix friction interactions or if the cell can achieve a net displacement after each bleb cycle in adhesion-independent conditions. Hence, a combination of biophysical modeling and cutting-edge experimental approaches is needed to elucidate T cell migration mechanisms and establish design criteria for next-generation immune engineering strategies to enhance T cell infiltration, migration, and sampling within the tumor mass.

Bleb nucleation^58–63^ and bleb growth^56,64–66^ have been studied theoretically using coarse-grained mechanistic models. In addition, a handful of amoeboid/bleb-based motility biophysical models have also been recently developed, where the intracellular and extracellular spaces were assumed to behave as viscous fluids.^49,50,67–79^ Most of these cell migration models assume that plasma membrane and cortex are a single composite material. Two of these studies, however, account for the mechanical interactions between the intracellular viscous fluid, the actin cortex, and the cell membrane.^67,68^ However, the model by Lim et al.^67^ does not satisfy conservation of momentum, since it does not account for the forces exerted by the cortex on the cytoplasm as it moves through the cortex to fill up the expanding bleb. Likewise, the model of Copos and Strychalski^68^ introduces a cortical viscoelastic force that depends on a reference cortical configuration as well as a steric interaction force with adjacent confining walls. Although the enforced no-penetration fluid flow condition at the boundaries ensures conservation of linear momentum in the fluid, the inclusion of these two forces does not guarantee that the total force exerted by the cell on the medium vanishes (i.e., does not ensure conservation of overall momentum). To address this gap, in the present study, we first develop a momentum-conserving mechanistic model of bleb-based cell swimming in a viscoelastic medium to assess whether bleb-producing cells such as T cells can mechanically swim in the absence of adhesions, then identify the key cellular components that regulate the motile capabilities of swimming cells, and quantify the theoretical maximum speed that a cell can achieve using this migration mechanism. Our initial focus on cell swimming is motivated by the persistent question of how T cells navigate through distinct microenvironments, ranging from lymph nodes to extracellular spaces of varying densities. We find that, while bleb-based cell swimming is theoretically possible, it does not seem to be sufficient on its own to explain the rapid T cell migration observed experimentally *in vivo*. Rather, our analysis supports an alternative hybrid model in which blebbing drives rapid protrusion extension, while subsequent weak and transient adhesion stabilizes the newly gained cell position. This proposed migration mechanism is reinforced by T cell experiments in 3D liquid-like solid (LLS) granular gels, demonstrating that adhesion-based forces are essential for effective migration. Our work provides key insights to mechanically control and potentially maximize the migration of blebby cells such as T cells, which can contribute to the design of tumor-stroma-targeting and cell-specific engineering strategies to enhance the intratumoral migration capabilities of antitumor immune cells.

## RESULTS

### Biophysical model of bleb-based cell swimming

**Figure 1.**
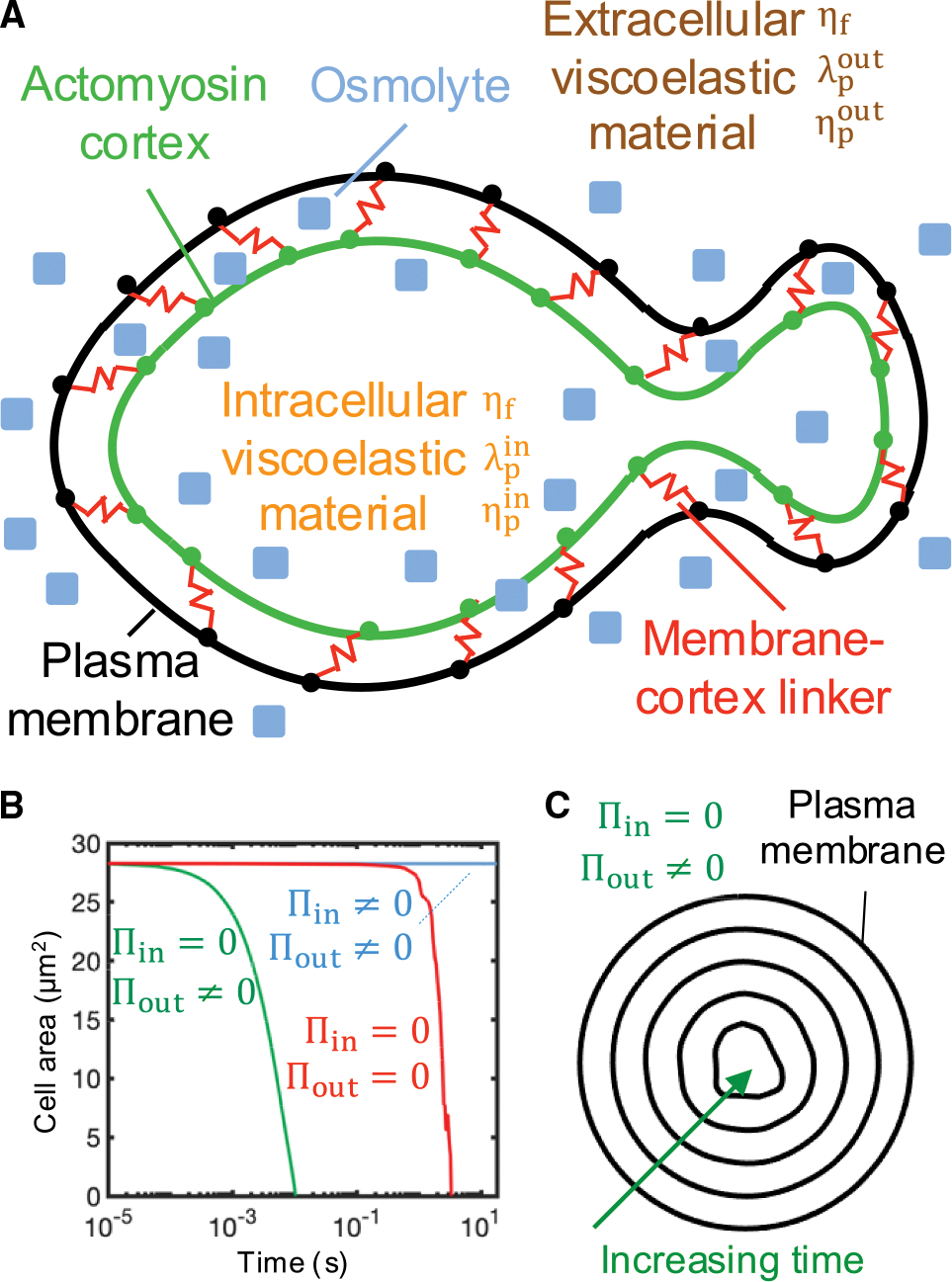
Intracellular osmotic pressure sets a stable cell size by counteracting cell shrinkage mediated by extracellular osmotic pressure and cortical tension (A) Schematic diagram of the bleb-based cell swimming model. The plasma membrane and actomyosin cortex are mechanically linked by reversible Hookean elastic linkers that stochastically associate and dissociate in a force-independent and -dependent manner, respectively. Intracellular and extracellular spaces are viscoelastic materials with associated fluid viscosity $\eta_{f}$, polymer viscosity $\eta_{p}$, and polymer stress relaxation time $\lambda_{p}$. Osmotic effects are included. (B) Time evolution of cell area for three different osmotic conditions. Intracellular osmotic pressure, Π_in_; extracellular osmotic pressure, Π_out_. (C) Cell shrinkage due to a reverse osmotic shock (Π_in_ = 0). Thus, inclusion of intracellular osmolytes is necessary to prevent cell collapse due to inward cortical tension and extracellular osmotic forces that tend to force water out of the cell.

We first aimed to investigate the cell migration capabilities of bleb-producing cells in an unbounded viscoelastic medium through the development of a 2D biophysical mechanistic model (see the supplemental information for a detailed description of the model). The cell is composed of two distinct structures: a cellular membrane and an actomyosin cortex (Figure 1A). Both are considered Lagrangian structures defined by a set of nodes that are connected to one another and the associated fluids as described below and in more detail in the supplemental information. As the cell migrates through the environment, both membrane and cortex structures transmit forces onto the surrounding medium, generating viscoelastic stresses. At the continuum level, conservation of mass and momentum on the incompressible viscoelastic fluid reads

(Equation 1) $$\nabla \cdot v_{f}(x,t)=0,$$

(Equation 2) $$\nabla \cdot \sigma_{tot}(x,t)+F_{tot}(x,t)=0,$$

(Equation 3) $${ℱ}_{\text{tot}\;}(x,t)={𝓕}_{m}(x,t)+{𝓕}_{c,\mathrm{drag}}(x,t)$$

where $v_{f}$ is the fluid velocity, $\sigma_{\mathrm{tot}}$ is the total stress, and ${𝓕}_{\mathrm{tot}}$ is the total force density on the fluid and equal to the sum of the membrane force density ${𝓕}_{m}$ and the cortical force density ${𝓕}_{c,\mathrm{drag}}$ generated in the intracellular medium. The total stress $\sigma_{\mathrm{tot}}=\sigma_{f}+\sigma_{p}$ is the sum of two contributions: a purely viscous stress $\sigma_{f}$ and a viscoelastic stress $\sigma_{p}$,

(Equation 4) $$\sigma_{f}=-\nabla p+\eta_{f}\left(\nabla v_{f}+\nabla {v_{f}}^{⊤}\right),$$

(Equation 5) $$\sigma_{p}=\frac{\eta_{p}(x,\;t)}{\lambda_{p}(x,\;t)}\left[\left(\frac{\kappa_{p}}{1\;-\;tr\left(\kappa_{p}\right)/L_{p}^{2}}\right)-I\right],$$

where we have chosen the finite extensible nonlinear elastic-Peterlin (FENE-P) constitutive relation for the stress to model the viscoelastic nature of the intracellular and extracellular spaces.^80^ Here, p is the hydrostatic pressure; $\eta_{f}$ and $\eta_{p}$ are, respectively, the fluid viscosity and the polymer viscosity; $\lambda_{p}$ is the polymer stress relaxation time; $\kappa_{p}$ is the conformation stress tensor; and $L_{p}$ is the polymer extensibility parameter, a measure of the maximum polymeric deformation. The time evolution of the conformation stress tensor $\kappa_{p}$ reads

(Equation 6) $$\begin{array}{ll} \frac{\partial \kappa_{p}}{\partial t}= & \frac{1}{\lambda_{p}}\left(I-\frac{\kappa_{p}}{1\;-\;tr\left(\kappa_{p}\right)/L_{p}^{2}}\right)-v_{f}\cdot \nabla \kappa_{p}\\ & \;+\;\nabla {v_{f}}^{⊤}\cdot \kappa_{p}+\kappa_{p}\cdot \nabla v_{f}. \end{array}$$

The first term on the right-hand side (RHS) of Equation 6 captures stress relaxation kinetics, the second term captures polymer advection, and the last two terms capture rotation and deformation of the polymeric material.

The cell membrane is an elastic structure subjected to tension, bending, and membrane-cortex adhesion forces, and the cortex is considered an actomyosin poroelastic structure that generates myosin-mediated forces that obey a linear force-velocity relationship and create tension in the cortical network.^61,81–83^ The actin cortex experiences drag forces as cytoplasmic material flows through the porous actomyosin network. Mass conservation of membrane-cortex linkers and actin and myosin in the cortex follows simple stochastic association and dissociation kinetics. No-slip between the plasma membrane and viscoelastic fluids is enforced. Furthermore, the model accounts for osmotic effects, which we found were essential for maintenance of cell volume. The intracellular osmotic pressure resists cortex and extracellular osmotic inward forces; all forces combined set cell size and shape. A hypothetical complete depletion of intracellular osmolyte would cause a sudden cell shrinkage within a few milliseconds, as the cortex would otherwise squeeze out all the intracellular water (Figures 1B and 1C). The model equations are solved using an immersed boundary method.^84^ The specific model parameter values used are summarized in Table S2, unless stated otherwise in the text/figure captions, and the code used for the T cell migration biophysical model is available in Alonso-Matilla et al.^85^ Additional details can be found in the supplemental information.

### Single-bleb formation, expansion, and retraction allow cells to swim in viscoelastic environments

**Figure 2.**
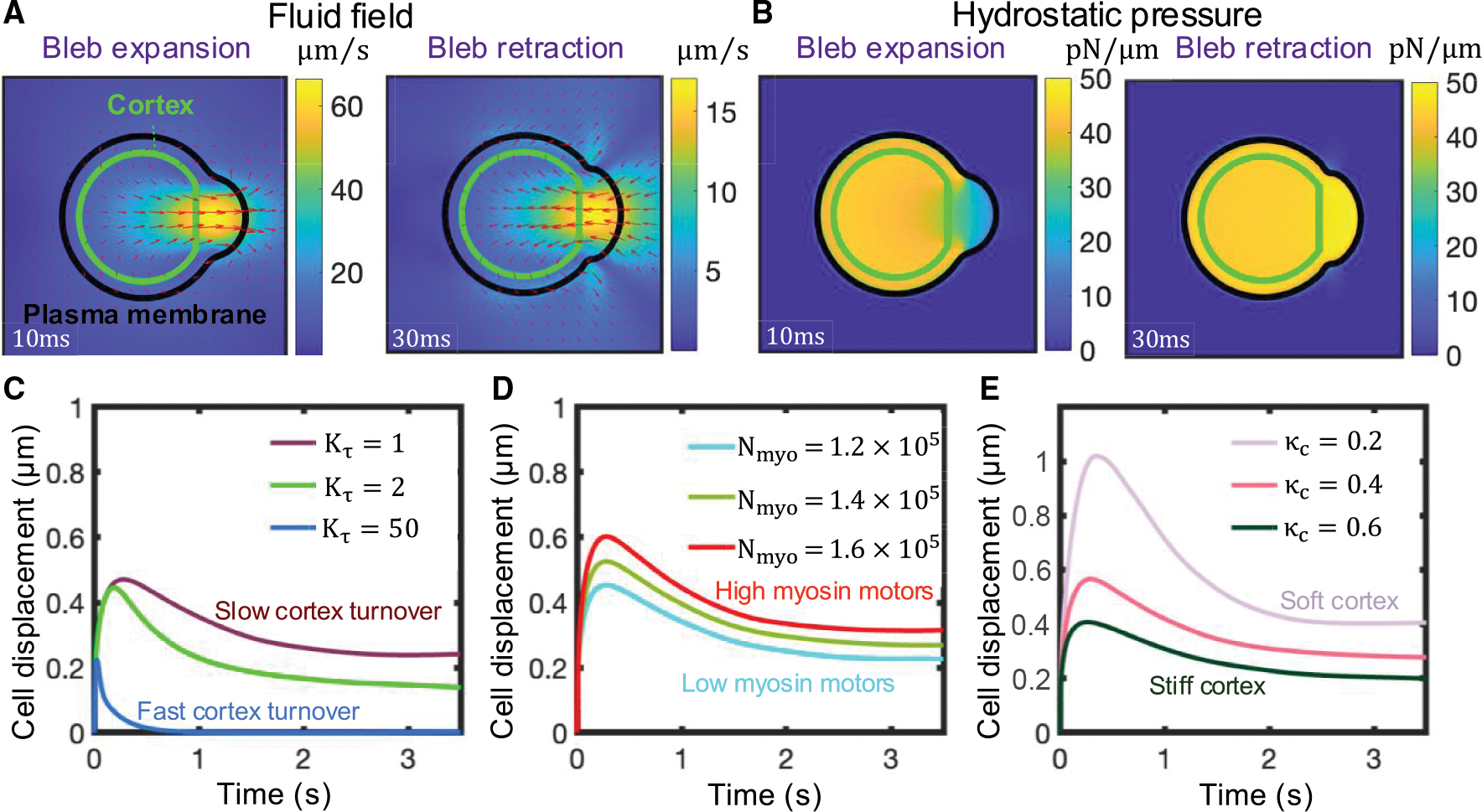
Cells can achieve moderate net cell displacements during a single bleb cycle in viscoelastic environments without adhesion-based forces, due to the differential shear rates associated with bleb growth and retraction phases (A and B) Snapshots of the magnitude of the fluid velocity and velocity vectors (A) and hydrostatic pressure (B) acquired at two time points during early bleb expansion and early retraction. $K_{\tau }=100$ for (A) and (B). (C–E) Time evolution of the cell centroid for three different values of the cortex turnover parameter $K_{\tau }$ (C), for three different values of the number of myosin molecules inside the cell N_myo_ (D), and three different values of the cortical stiffness per unit of actin parameter $\kappa_{c}$ (E). Greater cell displacements are achieved for slower cortical turnover kinetics and a more contractile and softer cortex. In (C)–(E), membrane-cortex linkers were broken by hand locally in a small region at time t = 0, mimicking a local downregulation of membrane-cortex linkers due to a chemical signal or force. Polymeric model parameter values: $\eta_{p}^{in}=1pN\cdot s\cdot \mu m^{-1},\;\lambda_{p}^{\text{in}}=100\;s,$ $\eta_{p}^{\text{out}}=1\;pN\cdot s\cdot \mu m^{-1}$, and $\lambda_{p}^{\text{out}}=10\;s$.

Using the above framework, we proceeded to study cell dynamics during a single bleb cycle by enforcing local membrane-cortex detachment. Membrane-cortex uncoupling is followed by a drop in local intracellular hydrostatic pressure, and cytoplasmic viscoelastic fluid material flows through the detached actomyosin meshwork from the center of the cell to the low-pressure region, mediating bleb expansion (Figures 2A, 2B, S1A, and S1B and Video S1). The poroelastic permeable cortex does not sustain pressure forces and moves retrogradely toward the cell center,^86,87^ with actomyosin contractile inward forces balanced by cytoplasm-mediated drag forces, which arise as cytoplasmic material flows through the porous actomyosin network (see Equation S16). Notice that, in the extreme case where the cortex-cytoplasm drag coefficient is extremely high, the actin meshwork moves along with the cytoplasm, preventing the cytoplasm from filling up the bleb. Eventually, cortex components that detach from the membrane disappear via turnover kinetics (i.e., first-order disassembly without concomitant assembly; see Equations S20 and S21). The bleb cycle ends by recruitment of new cortex constituents underneath the bleb membrane, which nucleates new actin filaments that recruit myosin motors that transmit inward forces to the plasma membrane, driving bleb retraction and cytoplasm from the bleb region to the center of the cell in a low intracellular hydrostatic pressure gradient phase (Figures 2A, 2B, and S2). We observe that the bleb area at the end of the cycle is primarily limited due to the increase in the local hydrostatic pressure from cortex re-formation rather than membrane tension (Figure S2). We observe that the plasma membrane follows similar trajectories during bleb expansion and bleb retraction. A forward movement of cytoplasmic material translocates the center of mass of the cell forward in the direction of the bleb during bleb growth, and a rearward movement of cytoplasmic material translocates the center of mass of the cell in the opposite direction during bleb retraction (Figures 2C, 2E, S3, and S4). According to Purcell’s scallop theorem, cell-shape-based swimming of microscale biological cells in a Newtonian fluid at low Reynolds number requires non-reciprocal body deformations to generate locomotion.^88^ Accordingly, we observe that net cell displacements at the end of the bleb cycle are minimal in Newtonian environments (Figure S2). This result is in contrast with the model by Lim et al.,^67^ which shows that individual blebbing events lead to significant net cell displacements. This discrepancy likely arises from a violation of momentum conservation in their model. However, reciprocal motion can lead to net cell motion in viscoelastic environments, as observed experimentally for single-hinge reciprocal microswimmers moving through shear-thickening and shear-thinning fluids.^89^ For physiologically relevant model parameters, cell displacements are larger during bleb expansion compared to those during bleb retraction due to the rapid and slow rates of bleb expansion and retraction phases, respectively. The cell shears its environment at distinct rates during both phases, resulting in significant net cell displacements (~ 0.4 *μ*m) during a single bleb cycle of ~2.5 s (Figures 2C and 2E) for a mean speed of ~9.6 μm/min. We can estimate the 3D effective cell motility coefficient as $D_{\text{cell}}=\frac{v_{\text{cell}}^{2}t_{\text{run}}}{4}$,^90^ where cells run at an effective velocity given by $v_{\text{cell}}=d_{\text{run}}/t_{\text{run}}$ during an effective runtime $t_{\text{run}}$. Given that reported blebbing frequencies are on the order of ~ 4 s^−1^,^86^ we assume that the time needed for the cell to translocate during each bleb cycle is the limiting factor in each cell run; thus $t_{\text{run}}\;\sim \;2.5\;s$ (see Figures 6A and 6B). Under these assumptions, $D_{\text{cell}}=d_{\text{run}}^{2}/\left(4t_{\text{run}}\right)=(0.4\;\mu m{)}^{2}/(4\times 2.5\;s)=0.96\;\mu m^{2}/min$. This value is an order of magnitude smaller than the *in vivo* motility coefficient of bleb-producing cells (~ 10 *μ*m^2^/min^21^), suggesting that the adhesion-free bleb-based cell migration mechanism is unlikely to be the mode of migration for T cells navigating through tissues. For non-physiological fast cortex turnover times, the timescales of bleb expansion and retraction are similar, preventing cells from achieving net cell motion during a single bleb cycle (Figure S4). Overall, our results indicate that net cell displacements can be achieved by bleb-based cell swimming in viscoelastic environments, but only at rates insufficient to explain rapid T cell motility *in vivo*.

### High intracellular hydrostatic pressure gradients and a softer cortex favor larger cell displacements during a bleb cycle

Because of uncertainty about our base parameter values, we explored how varying cellular conditions influenced cell displacements during an individual bleb cycle. For a given membrane-cortex detachment size, more contractile and/or softer actomyosin cortices favor larger cell displacements during bleb expansion (Figures 2D and 2E), resulting in greater overall cell displacements at the end of the bleb cycle. High cortical squeezing, mediated by a higher number of myosin motors in the cell, causes larger intracellular hydrostatic pressure gradients after membrane-cortex dissociation. This drives stronger cytoplasmic flows and results in larger bleb sizes and cell displacements during an individual bleb cycle (Figure 2D). A softer cortex gives rise to stronger intracellular flows (Figure S1A) and delays both intracellular hydrostatic pressure equilibration (Figure S1B) and the end of bleb expansion (Figure 2E). During this expansion phase, active stresses are concentrated at the cell’s rear and center, away from the actomyosin-free bleb region. The cortex, being a highly cross-linked actin network, allows these active stresses to propagate throughout the cell.^91^ As new cortex is recruited beneath the plasma-membrane bleb, it experiences retrograde pulling forces from the highly contractile regions away from the bleb, transmitting these forces to the plasma membrane at the leading edge. In cells with a softer cortex, this stress propagation is diminished, resulting in delayed bleb retraction and larger cell displacements at the end of a bleb cycle (Figure 2E). Interestingly, bleb expansion rates obtained from Figure 2 (~ 5 – 10 *μ*m/s) are at least an order of magnitude higher than diffusion-limited polymerization rates of monomeric G-actin into F-actin ~ 200 nm/s,^92–94^ which drive filopodial and lamellipodial extension, suggesting that maximal cell migration speeds might require bleb expansion dynamics rather than F-actin self-assembly-driven protrusions to drive rapid leading-edge advance.

The cell produces viscoelastic stresses on its surroundings as blebs expand/retract. The spatial structure of these stresses is examined in Figures S4A (inset) and S4D, where high polymer stresses concentrate at the front of the cell, providing an additional resistance to pressure-driven bleb expansion. This results in lower cell displacements at the end of the bleb cycle for extracellular materials with a low stress relaxation time $\lambda_{p}^{\text{out}}$. Cell displacements at the end of the bleb cycle are greater in low-density extracellular spaces (Figure S3A) and are nearly insensitive to extracellular stress relaxation times (Figure S3B). Low plasma membrane tension also causes maximal cell displacements in an individual bleb cycle (Figure S3C), as membrane tension builds up at the cell front during bleb growth, thus opposing further expansion (Figure S4D), and a softer membrane provides less elastic resistance to forward plasma membrane motion. Overall, our model results show that larger cell displacements during a single bleb extension-retraction cycle are favored by low-density extracellular spaces, providing novel insights into the mechanisms used by phenotypically amoeboid cells when navigating complex porous networks. Likewise, our model demonstrates that soft actomyosin cortices, high myosin motor levels, and softer plasma membranes also favor cell displacement, identifying intracellular factors that may be engineered to design cells that move better in mechanically heterogeneous complex environments.

### Sustained adhesion-free bleb-based cell swimming in Newtonian environments requires cortical contractility oscillations

**Figure 3.**
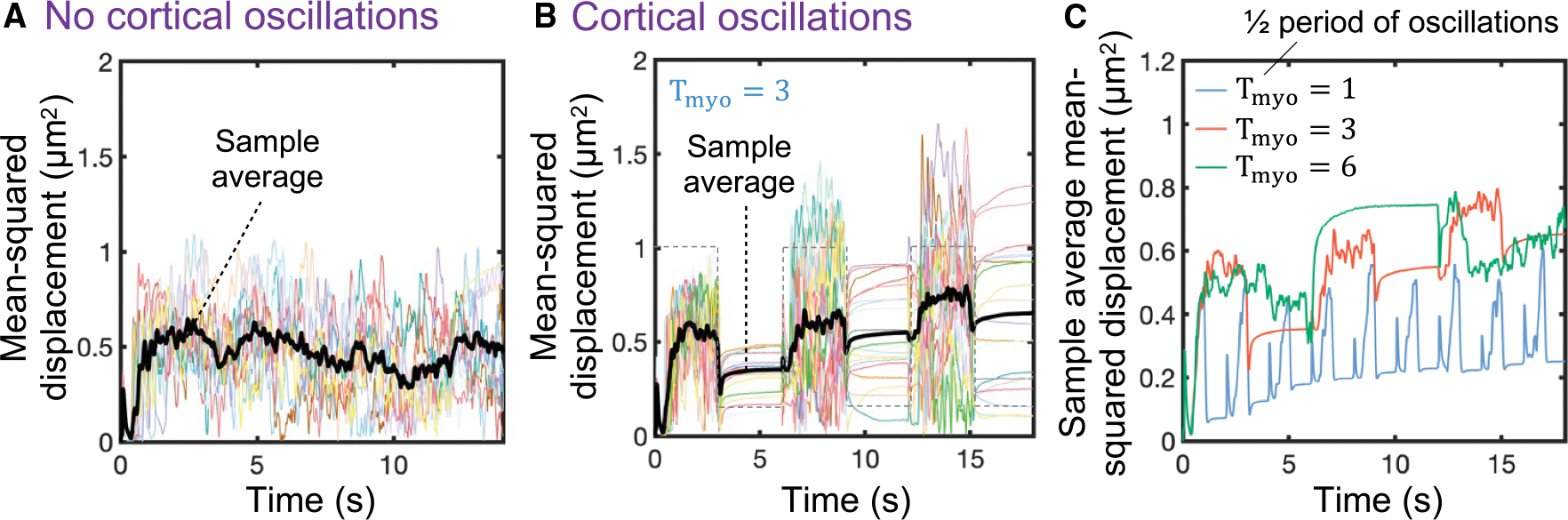
Cortical contractility oscillations are required for effective bleb-based cell swimming (A and B) Time evolution of mean squared displacement of 20 cells in the absence of cortical oscillations (A) and in the presence of cortical oscillations (B). The sample average over all cells (black line) increases over time for cells that produce cortical tension oscillations. Cells that do not produce cortical contractility oscillations are incapable of migrating effectively after the first bleb nucleation/expansion event. The gray dashed line in (B) indicates the waveform of the temporal oscillatory signal S_osc_. (C) Sample average mean squared displacement over time for three different periods (in seconds) of the cortical contractility oscillation signal. Intracellular and extracellular spaces are assumed to be Newtonian.

Since a single cycle of bleb extension-retraction results in minimal net cell displacement, we next explored whether bleb-producing cells can swim in adhesion-free conditions by producing bleb-based cell shape changes. To simulate more complex variations in the dynamics of cortex component amounts, we introduce stochasticity into the kinetics of membrane-cortex linkers, actin and myosin, where membrane-cortex linkers stochastically associate in a force-independent manner and unbind by force with an effective dissociation rate that increases exponentially with force according to Bell’s law^95^ (see the supplemental information for additional details). Hereinafter, we consider intracellular and extracellular spaces to be viscous fluids. We find that bleb-producing cells that maintain a consistently high contractility level cannot effectively migrate, as shown by the plateau reached by the sample average mean squared displacement-time curve after a few bleb nucleation events (Figure 3A and Video S2). The significant cell displacement achieved by the cells during the first bleb cycles suggests that cells could potentially migrate starting from a healed cortex with a fully pressurized intracellular cytoplasm. Therefore, we introduced a cortical contractility oscillator signal into the model to mimic cortical contractility oscillations^96,97^ and allow the cortex to recover and “heal” before stochastically initiating the next bleb cycle. We found that cells are capable of modest swimming by producing high-to-low cortical contractility oscillations, where a high cortical tension phase characterized by multiple bleb nucleation events is followed by an intracellular pressure buildup recovery phase at low cortical contractility, resulting in mean squared cell displacements that increase over time (Figure 3B). Intermediate frequencies of the cortical oscillation signal seem to maximize cell swimming migratory capabilities (Figure 3C). Overall, these results show that adhesion-free bleb-based cell shape changes combined with high-to-low cortical tensions are a possible strategy by which cells could move through biological tissues or fluids via a stochastic swimming behavior of repeated cycles of bleb-based protrusions driven by oscillations in cortical contractility. That is, our model predicts that amoeboid carcinoma and immune cell swimming is theoretically possible in Newtonian environments but is too slow to explain experimentally observed random motility coefficients associated with rapid T cell migration (~ 10 *μ*m^2^/min^46,98,99^).

### Maximal bleb-based migratory potential achieved for high myosin motors and a soft cortex

**Figure 4.**
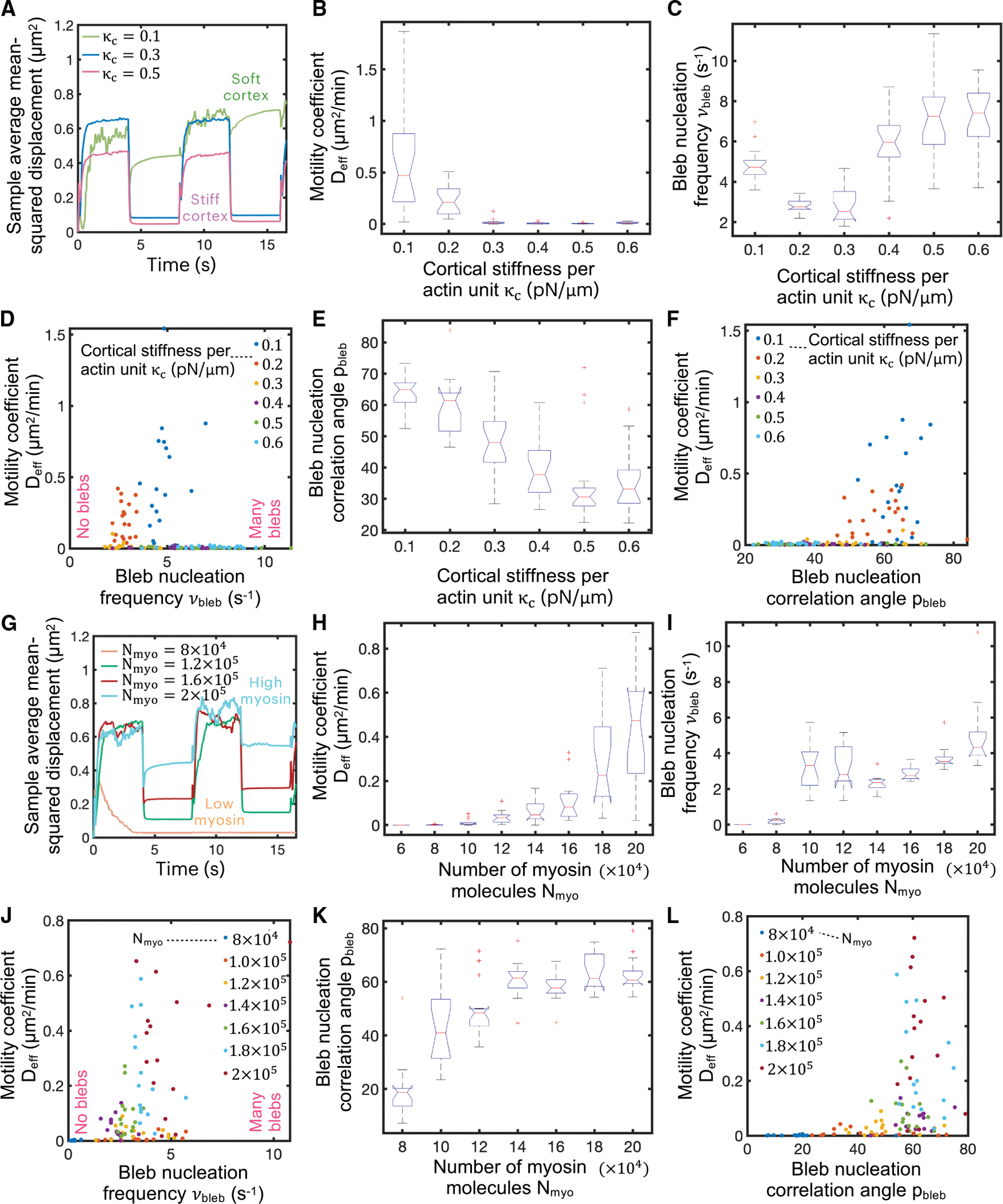
Swimming capabilities of bleb-producing cells can be modulated by cortical contractility and cortical stiffness (A) Time evolution of sample average mean squared displacement for four different values of the cortical stiffness per unit of actin parameter $\kappa_{c}$. (B and C) Effective random motility coefficient D_eff_ (B) and bleb nucleation frequency $\nu_{bleb}$ (C) as a function of $\kappa_{c}$. Cells swim more efficiently with lower cortex stiffnesses. The effective diffusion coefficient D_eff_ is extracted from the data by fitting a line constrained to go through the origin in the mean squared displacement-time plots over 16 s (D) Scatterplot showing the relationship between D_eff_ and $\nu_{bleb}$ for different values of $\kappa_{c}$. Intermediate bleb nucleation frequencies correlate with maximal random motility coefficients. (E) Polar angle between consecutive bleb nucleation events p_bleb_ as a function of $\kappa_{c}$. (F) Scatterplot showing the relationship between D_eff_ and p_bleb_ for different values of $\kappa_{c}$. Maximal random motility coefficients are observed for polar angles of ~60°–70°. (G) Time evolution of sample average mean squared displacement for number of myosin motors N_myo_. (H and I) Effective random motility coefficient D_eff_ (H) and bleb nucleation frequency $\nu_{bleb}$ (I) as a function of N_myo_. Cells swim more efficiently with high number of motors. (J) Scatterplot showing the relationship between D_eff_ and $\nu_{bleb}$ for different values of N_myo_. (K) Polar angle between consecutive bleb nucleation events p_bleb_ as a function of N_myo_. (L) Scatterplot showing the relationship between D_eff_ and p_bleb_ for different values of N_myo_. Maximal random motility coefficients are observed for polar angles of ~60°. Intracellular and extracellular spaces are assumed to be Newtonian.

To explore the optimum cellular conditions for maximal cell motility via the oscillatory bleb-based swimming mechanism identified above, we identify two important model parameters that strongly modulate cell motility: cortical stiffness and cortical tension. We find that a soft cortex is essential for cell motility (Figures 4A and 4B). Cortical stiffness per unit of actin, $\kappa_{C}$, higher than ~ 0.2 pN/*μ*m gives rise to extremely low random cell motility coefficients, as shown in Figure 4B. For an estimated number of actin units at each cortical node of ${\overset{‾}{n}}_{\text{act}}∼2\times 10^{4}$ (see supplemental information), our model predicts that a cell with a cortical stiffness greater than 4,000 pN/*μ*m cannot swim effectively. Quantitative analysis shows a biphasic behavior of bleb nucleation frequency $\nu_{bleb}$ as a function of cortical stiffness. When cortical stiffness is high, the cortex is unable to rapidly adjust to deformations in the plasma membrane. This frequently places membrane-cortex linkers under high tension, leading to frequent membrane-cortex linker ruptures and increased bleb nucleation frequencies. In the limit of low cortical stiffness, in-plane cortical motion caused by contractility gradients can also often subject membrane-cortex linkers to a high tensional state, causing frequent bleb nucleation events as well. Consequently, a minimum nucleation frequency is found at intermediate cortical stiffnesses, as depicted in Figures 4C and 4D. Intermediate bleb nucleation frequencies correlate with maximal cell random motility coefficients (Figure 4D). Whereas cells cannot swim under low-frequency conditions, since, effectively, cells nucleate a sequence of isolated bleb cycles, leading to no swimming, high-frequency-blebbing cells are not capable of successfully pressurizing their cytoplasm between consecutive bleb events, causing a decrease in their swimming propulsion efficiency. Intermediate blebbing frequencies are therefore ideal conditions for swimming, where the synchronized nucleation of multiple blebs indeed allows cells to break time reversibility and migrate with modest effectiveness (~1 μm^2^/min) in the absence of adhesion-based forces. Interestingly, the metastatic potential of prostate cancer cells has been reported to correlate with reduced F-actin and high blebbing frequency.^100^ The polar angle between consecutive bleb nucleation events (see supplemental information for further details), here referred to as bleb nucleation correlation angle p_bleb_, monotonically decreases with cortex stiffness, as shown in Figure 4E, with soft-cortex cells and stiff-cortex cells nucleating blebs at p_bleb_ ~ [60° – 70°] and p_bleb_ < 40°, respectively, as depicted in Figure 4F.

We additionally find that high cortical contractility is essential for bleb-based cell motility, with high-myosin-motor cells possessing a higher motility coefficient than low-myosin-motor cells, as depicted by the mean squared displacement curves, effective random motility coefficients, and wind rose migratory cell track (Figures 4G, 4H, and S5, respectively). Higher number of myosin motors increases blebbing frequency and bleb nucleation correlation angle (Figures 4I–4L). Maximal cell motility coefficients not only correlate with intermediate blebbing frequencies (as already discussed) but also with intermediate bleb-to-bleb polar angles, as shown in the scatterplots in Figures 4J and 4L. Maximal cell motility coefficients correlate with bleb nucleation frequencies of $\nu_{bleb}\;\sim \;4\;s^{-1}$ and bleb-to-bleb polar angles of p_bleb_ ~ 60°. Cells that nucleate sequential blebs with low correlation angles (p_bleb_ < 40°), i.e., blebs on top of blebs, approach geometric reciprocal motion, resulting in vanishing cell motility coefficients, whereas cells that nucleate sequential blebs too separated from one another spatially (p_bleb_ > 70°) lose directionality/cell polarization and do not create effective non-reciprocal plasma membrane deformations, and their migratory potential is penalized, as Figure 4L shows. Whereas slow turnover time of cortex components, low fluid viscosity, and low membrane tension favor bleb-based cell spreading, we have not observed any statistically significant difference in migration capabilities for the different values explored for cortex porosity, as depicted in Figure S7. We hypothesize that the actomyosin cortical meshwork hinders cytoplasmic flows during both bleb expansion and bleb retraction, and so the slowing of cytoplasmic flows during both phases cancels out, and its effect on bleb-based cell motility becomes insignificant. Overall, our model results show that cells with a soft cortex and elevated cortical tension exhibit more efficient motility by extending large bleb protrusions and the precise spatiotemporal nucleation of blebs, suggesting that engineering therapeutic T cells to possess increased cortical tension and/or a softer cortex would favor bleb-based T cell movement in complex environments. Notably, our cortical tension result is consistent with recent experimental findings where high contractility pushed adhesive T cells into a faster amoeboid phenotype.^21^

### Blebs nucleate in regions of high cortical tension, low membrane-cortex linker density, and high intracellular osmotic pressure

**Figure 5.**
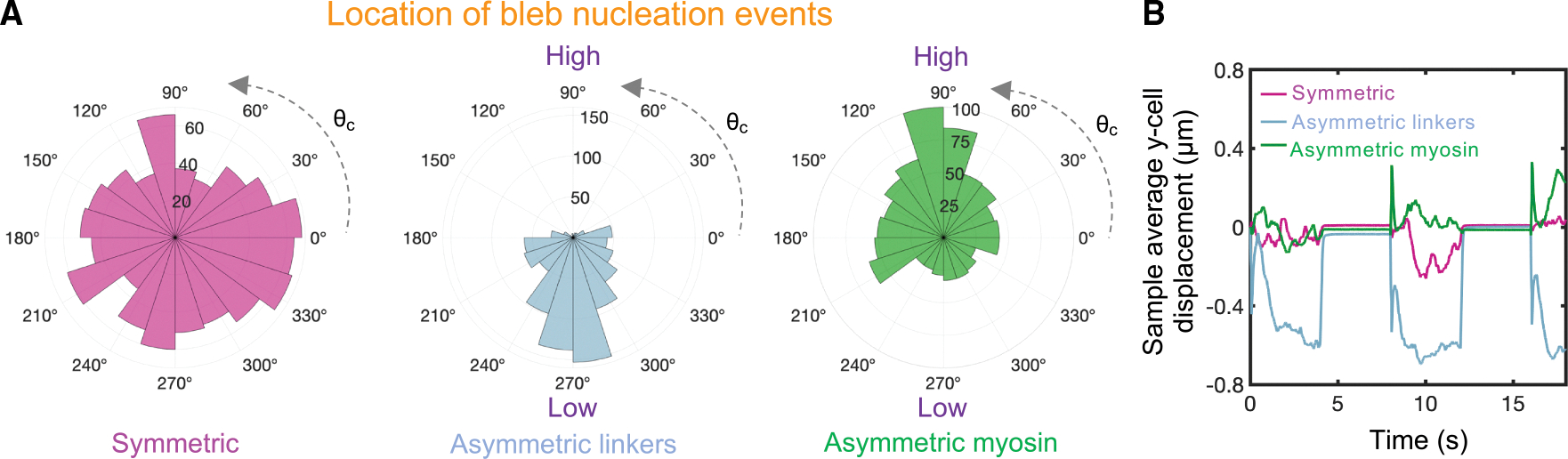
Blebs nucleate in regions of high myosin and low membrane-cortex linker levels (A) Polar distribution of bleb nucleation events for three different conditions: uniform association of membrane-cortex linkers and myosin, $k_{adh_{eff}}^{on}=k_{adh}^{on},k_{myo_{eff}}^{on}=k_{myo}^{on}$ (red violet); asymmetric kinetic association rate constant of membrane-cortex linkers, $k_{adh}^{on}=k_{adf}^{on}\left(1+0.2\;sin\;\theta_{c}\right)$ (blue); and asymmetric kinetic association rate constant of myosin, $k_{{myo}_{\text{eff}}}^{on}=k_{myo}^{on}\left(1+0.2\;sin\;\theta_{c}\right)$ (green). Here, $\theta_{c}$ is the polar angle associated with each cortical node. (B) Time evolution of sample average vertical cell displacements for the three different conditions. For the chosen parameter values, larger blebs are formed in the asymmetric linker configuration, resulting in larger displacements during the high cortical contractility phase. Intracellular and extracellular spaces are assumed to be Newtonian.

We next investigated the role of spatial variations in mechanical factors that potentially control the location of bleb nucleation sites and thus bleb-based cell directionality. Bleb nucleation events are initiated by a local decrease in membrane-cortex cohesion. We explored three different scenarios: spatially dependent cortical myosin recruitment, spatially dependent membrane-cortex linker recruitment, and asymmetric distribution of electro-osmotic pumps within the plasma membrane. Blebs preferentially form in regions of high cortical contractility (high myosin) and low membrane-cortex linker levels, as shown in Figure 5A, and in agreement with experimental observations.^22,101,102^ Linkers preferentially reach a high tensional state in areas of myosin enrichment, as they are subjected to higher cortical inward forces that eventually cause them to dissociate by force in a runaway process, giving rise to local membrane-cortex detachment and bleb formation. As the bleb enlarges, the membrane-cortex detachment size keeps increasing due to the ongoing rupture of membrane-cortex linkers, while a new cortex forms beneath the plasma membrane. Concurrently, the expansion of the bleb shifts the cortical force at its neck from inward to outward, reducing the tensional state of linkers and effectively preventing further detachment of the membrane from the cortex. In a similar way, linkers located in low-linker-density regions are overpowered by high cortical loads, making them more susceptible to failure. The highly polarized orientational distribution of blebbing events forces cells to extend protrusions along one axis, restricting their motion to only 1 degree of freedom and in turn impeding their migration, as shown in Figure 5B and consistent with the scallop theorem.^88^ Therefore, enforcing the formation of blebs in a small subregion of the cell turns out to be a poor cell-shape-change-based swimming strategy. Linker failure is also exacerbated in high intracellular osmotic pressure regions (Figure S8). The highly porous cortex does not sustain net osmotic forces; therefore, an increase in local intracellular osmotic pressure causes membrane outward motion, straining linkers, which results in a cascade of linker failures and bleb formation. This finding is consistent with the study by Yoshida and Soldati,^103^ which showed that low-osmolarity medium triggers blebbing, highlighting the importance of osmotic stresses in blebbing. Overall, our model results show that a competition between membrane-cortex cohesion and a combination of osmotic and myosin-driven cortical forces can control the location of bleb formation sites, again identifying potential cell engineering design strategies to alter and control cell protrusion dynamics to enhance motility.

### Rapid cell migration requires cell-matrix adhesion forces and is potentially enabled by a hybrid bleb- and adhesion-based mode of migration for optimum cell motility

In order to analyze whether bleb-based swimming is a feasible mechanism that explains observed T cell motility *in vitro* and *in vivo*, we explored if blebbing alone can mediate rapid cell migration in the absence of adhesion-based forces or if it requires adhesion interactions with the environment for optimal motility. We found that bleb-based coordinated cell-scale deformations are inefficient and lead to maximal migration motility coefficients of ~ 1.5 *μ*m^2^/min (Figure 4), an order of magnitude lower than reported *in vivo* cell motility coefficients of bleb-producing cells such as T cells (~ 10 *μ*m^2^/min^21^), which display much faster speeds than those achieved by mesenchymal cells (~ 1.5 *μ*m^2^/min for U251 glioma cells^104^), suggesting that swimming is not the primary T cell movement mechanism.

**Figure 6.**
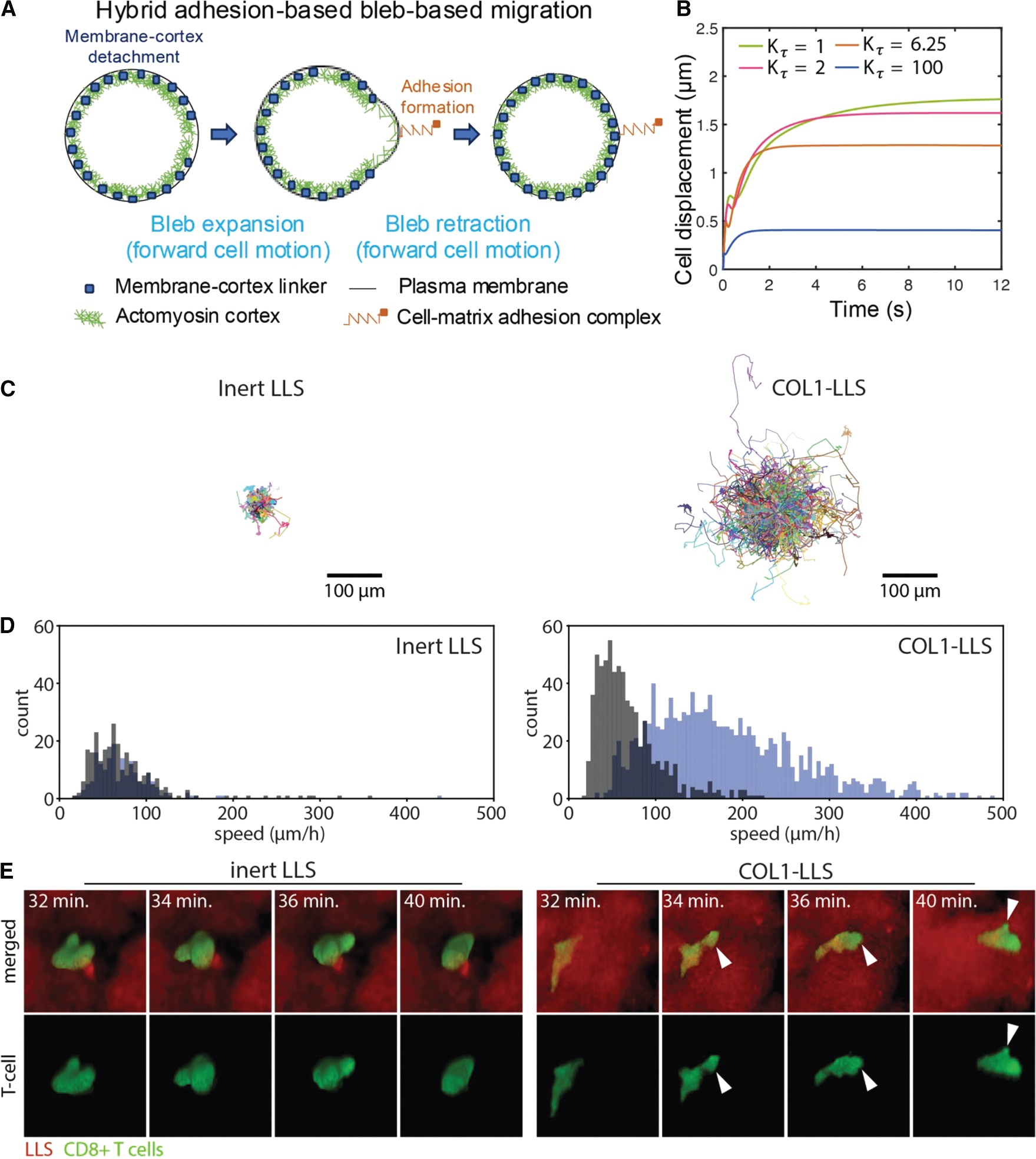
Biophysical model and experiments show that cell-extracellular matrix adhesion is required for rapid T cell motility (A) Visual representation of one complete cell migration cycle in the hybrid adhesion-based bleb-based mode of migration. (B) Model prediction of the time evolution of the cell centroid for four different values of the cortex turnover factor $K_{\tau }$. Larger cell displacements are achieved for slow cortex turnover times. Physiological cortex turnover times correspond to $K_{\tau }\;\sim \;1$ (see Table S1). (C) Projected *x-y* migration trajectories of T cells in inert LLS microgels (left) and COL1-LLS microgels (right). T cells exhibit faster and more extensive migration in the presence of collagen. (D) Histograms of walking speeds for T cells migrating in inert LLS and COL1-LLS. Speed was defined for each cell as the cell diameter divided by the time required to travel a distance equal to one cell diameter. Mean walking speed was calculated separately for two subpopulations: cells shown in blue reached a mean squared displacement (MSD) greater than one cell diameter squared within 3,000 s of lag time, while cells shown in black did not meet this threshold. See Video S4 for experimental data corresponding to (C) and (D). (E) Selected confocal time-lapse images showing a single T cell migrating through the 3D interstitial space between microgel particles in inert LLS (left) and COL1-LLS (right). See Videos S5 and S6 for the full time-lapse sequences. White arrow indicates the bleb position.

To address this discrepancy between maximum swimming speed and well-documented T cell motility coefficients *in vitro* and *in vivo*, we investigated the effects of cell-environment adhesion interactions on the migration capabilities of bleb-producing adherent cells by constructing a simple hybrid adhesion-based, bleb-based cell migration biophysical model (Figure 6A, see the supplemental information for a detailed description of the model). Our findings indicate that adhesion interactions with the environment significantly increase cell migration speeds, with cells being capable of translocating approximately up to d_run_ ~ 1.8 *μ*m during an isolated bleb cycle of 9 s (Figure 6B and Video S3), for a mean speed of ~12 μm/min. In this mode of migration, we observe that blebbing allows cells to push their cell front forward at a very fast rate (~ 10 *μ*m/s), much faster than F-actin polymerization rates and consistent with experimental observations,^86^ followed by formation of focal adhesions at the cell front, which prevents cell rearward motion during bleb retraction and mediates subsequent traction force generation and fast forward cell translocation. Our model shows that bleb-producing adherent cells migrate more effectively for slow cortex turnover times (Figure 6B) and under high cortical tension conditions (Figure S9A). Interestingly, while a softer cortex favors greater cell displacements during bleb expansion, a stiffer cortex causes larger cell displacements during bleb retraction, with the highest net cell displacements occurring at intermediate cortical stiffnesses (Figure S9B). Repeated cycles of adhesion-based blebbing dynamics can lead to diffusive cell behavior, assuming that bleb nucleation sites are independent of the location of previous bleb nucleation events. We can again estimate the 3D effective cell motility coefficient associated with this hybrid migration mechanism as $D_{\text{cell}}=v_{\text{cell}}^{2}t_{\text{run}}/4=60\times (1.8\;\mu m{)}^{2}/(4\times 9\;min)=21.6\mu \;m^{2}/min$. This value is comparable to the in vivo motility coefficient of bleb-producing cells, suggesting that the hybrid cell migration mechanism is likely the mode of migration for T cells navigating through tissues.

To experimentally test our model prediction that cell-matrix adhesion forces are essential for rapid T cell motility, we employed established LLS microgels with bioconjugated type 1 collagen (COL1)^105^ and examined human CD8^+^ T cell invasion in 3D COL1-LLS environments.^106^ Collagen conjugated to the surface of the microgels promotes cell adhesion and facilitates migration through the interstitial spaces between the microgel particles. This platform allows us to decouple MMP-dependent factors and specifically investigate the contribution of adhesion to 3D cell migration. The figure/supplemental videos demonstrate that T cells are capable of squeezing through narrow interstitial spaces. However, this migration is significantly enhanced in the presence of adhesion cues on the microgel surface compared to T cell migration in inert LLS, where cells lack the ability to adhere to their surroundings. Taken together, our results demonstrate that T cell migration is significantly faster under adherent conditions in COL1-LLS compared to non-adherent conditions in inert LLS (Figures 6C and 6D), supporting the presence of cell-environmental adhesion forces in promoting rapid motility.

Additionally, our model predicts that, in cells employing a hybrid adhesion-bleb-based migration strategy, cell displacements remain unaffected by the stiffness of the substrate, as shown in Figure S9C. This finding aligns with experimental observations of T cells on quasi-3D elastic platforms.^21^ In these experiments, cells treated with nocodazole, a microtubule destabilizer, exhibited high contractility and an amoeboid-like morphology. These cells showed a migration potential that was independent of the rigidity of the nanotexture (see Figure 2C in Tabdanov et al.^21^), potentially suggesting that nocodazole-treated highly contractile T cells might use a hybrid bleb-adhesion-based migration mode. The observed insensitivity of cell migration speed on substrate rigidity among these cells starkly differs from the behavior observed in the adhesion-based motor-clutch migration mode, where migration of highly contractile cells is sensitive to the stiffness of the substrate.^25^ Crucially, the effectiveness of the hybrid adhesion-bleb migration mode hinges on the predominant formation of cell-matrix adhesions at the leading edge of the cell (Figure S9D). If cell-matrix adhesions are largely formed at the rear of the cell, cell movement is predicted to be significantly impaired (Figure S8D). Increased membrane tension at the leading edge (Figure S4D) may locally activate adhesion molecules,^107^ such as integrins, thereby promoting a biased formation of cell-matrix adhesions at the front. This spatial bias would enhance directed migration by coordinating hydrostatic pressure gradients with traction forces. We also note that enhanced traction forces mediated by actin polymerization forces have not been considered in this analysis, but it is predicted that they would enhance significantly the cell migratory potential of adherent blebby cells. However, how cells coordinate the spatiotemporal assembly and disassembly of focal adhesions, actin polymerization, and traction forces with blebbing remains to be determined.

In summary, our findings uncover potential mechanisms governing the remarkable ability of T cells to rapidly navigate through tissues, suggesting that cell-matrix adhesion enables these cells to generate rapid and sustained motility within complex microenvironments. Our model and experimental results do not support an adhesion-free swimming strategy relying solely on changes in cell shape, specifically, through the formation of protruding blebs. Instead, this mechanism appears to allow them to slowly explore/sample the surrounding microenvironment prior to robust directed movement. Importantly, our findings suggest that fast migration requires the presence of cell-surrounding matrix adhesion interactions. The synergy between fast forward plasma membrane motion driven by bleb expansion, followed by cell-matrix adhesion formation and subsequent traction force production characteristic of mesenchymal migration, likely optimizes T cell motility.

## DISCUSSION

We developed a mechanistic model of bleb-based cell motility and conducted T cell migration experiments in soft, LLS granular media under both adherent and non-adherent conditions. Our work elucidates the potential physical principles and molecular components that modulate T cell migration, examines whether adhesion is required for rapid motility, and explores how perturbing these molecular regulators can modulate their migration potential. The amoeboid cellular phenotype has been associated with immune cell migration^21,22,108^ as well as with multiple cancer cells displaying epithelial-to-amoeboid transitions with enhanced cell invasion, immunosuppression, stem cell potential, and metastatic capabilities.^109–113^ Although the developed model is aimed at providing insights to effectively engineer therapeutic T cells with enhanced migration capabilities, the model’s applicability extends broadly to all bleb-producing cells, including amoeboid cancer cells, with a major goal being to induce fast amoeboid T cell migration and target/inhibit amoeboid cancer cell migration. While our model addresses crucial aspects relevant to this research area by elucidating the most plausible mechanisms of fast T cell migration, consistent with experimental data and within the constraints of current modeling capabilities and knowledge, it does not capture every complex detail inherent in real cells. Integrating every molecular constituent and biological aspect into our biophysical model is beyond practical reach. Missing explicit intracellular and extracellular factors include microtubules, septins, aquaporins, and cytokines, which are known to influence cortex behavior, the functionality of cell adhesion molecules, and the overall migration of T cells.^21,114–116^ According to Tabdanov et al.,^21^ microtubules play a more significant role in regulating GTPase RhoA signaling than serving as direct mechanical elements. GEF-H1 associates with polymerized microtubules, and upon depolymerization, GEF-H1 is released, increasing its guanosine nucleotide exchange activity. This leads to higher RhoA activation and increased cortical contractility.^21,96^ Our model indirectly incorporates the effect of microtubules on T cell migration by simulating this global/local effect on actomyosin contraction (Figures 2D and S9A). Additionally, our model does not account for the role of the nucleus, which acts as a physical barrier and completely arrests T cell migration through interstitial pores smaller than 7 *μ*m^2^ when matrix metalloproteinase-independent pathways are involved.^117^ While the nucleus is thought to impede migration by resisting cytoplasmic flows, due to both steric hindrance and mechanical resistance through its binding to the cytoskeleton, deformation of the nucleus under confinement may also activate mechanotransduction pathways that enhance actomyosin contractility.^118,119^ Although such effects could influence migration speed, explicitly modeling the nucleus would require assumptions about its material properties and coupling to the cortex that remain speculative. We therefore chose to focus on well-characterized mechanical features while acknowledging the nucleus as an important future direction. Overall, we expect that, mechanically, the nucleus will act to slow down migration, and therefore our analyses omitting the nucleus can still be accurately regarded as theoretical upper limits.

Another important factor not explicitly captured is the effect of geometric confinement and wall-like boundaries. *In vivo*, T cells frequently encounter structures such as matrix pores or surrounding cells, which may impose physical constraints that alter migration mechanics. Confinement may facilitate directional bleb expansion along narrow channels but can also hinder migration during bleb retraction through non-specific frictional interactions, particularly involving the larger surface area of the cell body. This is consistent with our finding in Figure S9D, which shows that the hybrid adhesion-bleb migration mode is most effective when cell-matrix adhesions are concentrated at the leading edge. Under confinement, this spatial distribution may be disrupted, potentially slowing net migration. Simulating these interactions would require incorporating boundary forces at high spatial and temporal resolution while conserving momentum, a challenge that remains computationally infeasible in our current framework. As discussed in our recent review,^120^ the physical nature of these weakly adhesive interactions is still poorly defined and may involve weak clutches mediated by non-specific van der Waals forces, hydrogen bonds, ionic interactions, or components of the glycocalyx. Future modeling and experimental efforts will be needed to fully understand how confinement shapes T cell motility. In addition, our model can be extended in the future to incorporate more realistic features such as aquaporin dynamics, local changes in actin cortex permeability, and electro-osmotic effects. These additions, however, were beyond the scope of the present study.

Our model results indicate that sustained adhesion-free bleb-based cell motility requires oscillatory cortical forces in Newtonian environments, where cells alternate between a high-contractility motility phase and a low-contractility pressure buildup phase. These pulsatile actomyosin forces, which can arise from different mechanochemical sources, including calcium signal fluctuations,^97,121^ myosin phosphorylation-based biochemical oscillators,^122,123^ and ERK-based mechanochemical signaling, among others, do not seem to be essential in bleb-based cell swimming only but also in other biological contexts, including adhesion-based cell migration,^96^ compaction,^124^ cell intercalation^97^ and tissue integrity^123^ during morphogenesis, and temporal regulation of actin polymerization and vesicle exocytosis.^121^ Future theoretical work could incorporate existing reaction-diffusion models^125–127^ into our bleb-based cell migration framework to more explicitly capture the biochemical mechanisms underlying contractility oscillations. For example, integrating models of oscillatory calcium dynamics could help elucidate how biochemical calcium signaling networks contribute to the temporal regulation of cortical tension and its impact on cell motility.

Repeated sequences of a single bleb nucleation event, bleb expansion, cortex recruitment, and bleb retraction do not cause sustained cell swimming in Newtonian fluids, since the plasma membrane follows similar paths during bleb expansion and retraction. However, we show that individual blebs in viscoelastic environments or spatiotemporal synchronization of multiple bleb nucleation events in Newtonian environments allows cells to break time reversibility and effectively migrate in the absence of adhesion-based forces (i.e., swim), albeit at rates well below those exhibited experimentally by T cells. Additionally, our results indicate that coordinated cell-scale deformations driven by blebs are inefficient and result in maximal motility coefficients of ~ 1.5 *μ*m^2^/min, an order of magnitude lower than those reported by bleb-producing cells *in vivo* (~ 10 *μ*m^2^/min^21^). In such poor swimming conditions, interstitial flows would dominate in certain tissues over the inherent swimming motion of cells, rendering cell swimming less relevant to cell translocation.^128,129^ Our analysis gives a new physical perspective of the mechanism underlying potential swimming of bleb-producing cells and provides a strategy to modulate cell swimming potential by targeting the cortex, plasma membrane, and extracellular environment.

Other possible adhesion-free cell swimming mechanisms rely on peristaltic waves,^130^ osmotic pressure gradients,^131^ Marangoni stresses,^49,50^ or slippage between plasma membrane and surrounding fluid,^132^ possibly caused by mechanical coupling of transmembrane proteins, powered by F-actin retrograde flows, with the surrounding fluid.^40,48^ Although these migration models are not analyzed within our theoretical framework, our experimental results already indicate that adhesion-based migration is inherently faster and more efficient than non-adhesive modes. Reversat et al.^44^ proposed an alternative mechanism for adhesion-free migration, suggesting that T cells can navigate through serrated microfluidic channels without relying on adhesion or friction-based forces. Their theory posits that cell migration is driven by intracellular pressure gradients generated by retrograde flows and the curvature of actin flows caused by the channel wall topography. While their experimental results show an intriguing dependence of cell migration speed on wall topography, their proposed migration model contradicts established physical laws. The authors claim that the local force exerted by intracellular hydrostatic pressure gets directly transmitted to the channel walls. According to their theory, this pressure force, acting perpendicular to the walls and summed across the cell’s surface, generates a net hydrostatic force serving as a propulsion mechanism that drives the cell in the direction opposite to the actin flows. Their interpretation overlooks critical physical aspects of plasma membranes. A local increase in intracellular hydrostatic pressure will induce a small efflux of water, drawing the plasma membrane inward, away from the channel walls and thus not transmitting any hydrostatic force to the walls, as the authors claim. In addition, it is unclear whether T cells migrated through these microchannels in the complete absence of adhesion/friction forces, as the authors claim, due to the technical challenge of completely blocking non-specific adhesions. A plausible interpretation of their experimental findings, consistent with our model results, is that the magnitude of the adhesion/friction-based forces within the cellular environment (i.e., channel walls) may depend on its topographical features. We note that weak adhesions that last only a few seconds are sufficient in our model to enable fast migration in the hybrid amoeboid-mesenchymal mode.

Although cell signaling might play an important role in the coordination of multiple blebbing events, cells can orchestrate blebbing and control bleb frequency and bleb-to-bleb distance by temporal modulation of cortical tension and stiffness, where high tension and soft cortex favor optimum conditions. To evaluate whether higher speeds could be achieved under non-adhesive conditions, we explored non-physiological parameter combinations, such as extremely soft cortices paired with high myosin levels. While these conditions transiently increased speed, they rapidly caused mechanical instabilities, including cortical rupture and loss of shape integrity, making them unsuitable for sustained motility. Notably, the observed increase in speed with higher cortical tension aligns with experimental findings that associate elevated contractility with rapid amoeboid-like migration.^5,24,133^ Motor activity levels can be modulated by the internal cellular state as well as by environmental cellular conditions. As an example, nuclear deformation mediated by enhanced cellular confinement can activate mechanotransduction pathways^118,119^ that enhance actomyosin contractility and therefore would be predicted to increase cell swimming potential. Whereas poorly contractile cells nucleate blebs at low frequencies and in regions near recent bleb nucleation sites, highly contractile cells nucleate blebs at ~ 4 Hz and cause membrane-cortex detachment at bleb-to-bleb separation polar angles of ~ 60°. These blebbing frequencies and bleb-to-bleb separation distances correlate with maximal cell motility coefficients.

In addition, our model shows that blebbing events are expected to be favored in cell regions of high cortical forces, low membrane-cortex adhesion, and local intracellular osmotic pressure. This is consistent with experimental data, where blebbing was less frequent in the ezrin/radixin/moesin-rich uropod-like structure of cells^28,134–136^ and favored in calcium-dependent high myosin contraction regions at the leading edge of cells.^13^ Chemoattractants, which have also been associated with bleb polarization,^137–139^ potentially modulate some of the three driving factors mentioned above. Interestingly, we find that unidirectional bleb-based cell swimming is not possible via highly polarized bleb nucleation events, strongly suggesting that persistent directional cell migration, observed in different confined systems,^11,26,43,140^ might require the presence of cellular frictional/adhesive forces with the environment, as assumed in the hybrid amoeboid-mesenchymal model.^39,44^

Our findings indicate that T cells may use a hybrid migration strategy, integrating bleb formation with cell-matrix adhesion to optimize motility. Our model is supported by experimental T cell migration data, which demonstrate that T cells move significantly faster in microgels functionalized with COL1 than in inert gels. In our system, collagen monomers are present only on the surface of the microgels at the cell-gel interface. The 3D platform is composed of polyacrylamide microgel ensembles, where the interstitial spaces between particles provide physical pathways for cell migration. This approach uniquely enables independent control over structural parameters, including particle size, water content, stiffness, topography, topology, and permeability, while enabling separate modulation of COL1-mediated adhesion, as previously described.^141,142^ For this study, we employed two conditions: collagen-conjugated LLS (COL1-LLS) and inert, unconjugated LLS microgels. The inert gels, lacking adhesion molecules, have been shown to create frictionless environments,^141^ offering the most direct and effective negative control for adhesion-based migration. We have demonstrated that fully hydrated polyacrylamide exhibits a negligible friction coefficient^141–143^ and therefore does not provide adhesive cues to migrating cells. In contrast, COL1 bioconjugation introduces adhesion sites that support cell binding and promote migration along interstitial paths.^105,106^ Accordingly, this experimental design provides a robust framework to distinguish between adhesion-dependent and adhesion-independent T cell migration.

Unlike in bleb-based cell swimming, the cell advances in the same direction during both bleb expansion and retraction phases in this proposed hybrid mode of migration, achieving speeds that are comparable to physiological T cell migration speeds. Our results indicate that adherent blebby T cells can exhibit a highly migratory phenotype by increasing their contractility and fine-tuning their cortical stiffness to intermediate values. This mode of migration is consistent with a recent study that shows that T cells push and pull on the extracellular matrix and require adhesion-based forces to migrate through *in vitro* systems.^52^ Recent experimental observations additionally support this hybrid mode of migration. For instance, melanoma cells exhibit blebbing while moving through soft collagen matrices, pushing collagen aside at the leading edge during bleb expansion and pulling it in during bleb retraction via adhesion,^144^ as in our hybrid model. Similarly, rapid β1-integrin-dependent bleb-based migration of breast cancer cells involved extracellular matrix reorganization at membrane blebs^145^ with integrin clustering occurring at these sites. These findings align with the fast hybrid adhesion-based bleb-based migration mechanism, which relies on the predominant formation of cell-matrix adhesions at the cell’s leading edge. It is also consistent with another cell migration study where recruitment of the branched actin nucleator Arp2/3 frequently follows membrane-cortex detachment, depletion of the ERM membrane-cortex linker ezrin, and protrusion expansion.^102^ In some instances, however, cell-extracellular matrix adhesion formation and blebbing events anticorrelate in space, potentially suggesting the presence of F-actin assembly driven protrusions or pressure-only driven protrusions.^146^ Interestingly, recent work by Te Boekhorst et al.^147^ demonstrated that cancer cells can exhibit mixed migration modes combining blebbing and actin-based protrusions, with amoeboid phenotypes prevalent under low-adhesion conditions, consistent with effective migration using a hybrid mesenchymal-bleb-based mechanism.

Recent modeling results also suggest that cortical squeezing at the cell rear combined with focal adhesion formation at the cell front is a fast and robust cell migration strategy.^148^ It is unclear, though, how blebbing, actin polymerization forces, traction forces, and spatial dynamics of focal adhesion assembly and disassembly integrate all together in this model.

Experimental studies in mesenchymal cells that use the motor-clutch model showed that clutch-extracellular matrix binding and initial force transmission can occur within 1–10 s.^149,150^ This timescale is comparable to the duration of a typical bleb cycle (Figure 6B, green curve). In the context of bleb-driven migration, actin cortex reassembly begins rapidly following bleb expansion, and myosin II recruitment typically occurs within 5–10 s.^151,152^ Therefore, effective adhesion-mediated migration during bleb retraction does not require adhesion formation to be instantaneous after actin recruitment but rather needs only to occur within seconds following cortex assembly, approximately within the same timescale as myosin recruitment. In our hybrid bleb-based mesenchymal model, cell-matrix adhesion is assumed to form at the end of bleb expansion, a timing that is consistent with this sequence of events. This assumption of immediate adhesion and traction after expansion defines an upper bound on cell migration speed in our model. Future extensions of our model will aim to explicitly incorporate actin polymerization-driven protrusions and dynamic adhesion kinetics to more fully characterize the parameter space and transitions between swimming, bleb-based adhesion-dependent migration, and mesenchymal-like migration.

Although friction has been associated with the major factor explaining cell migration in passivated microfluidic devices,^11,140^ it remains uncertain whether cellular interactions with the extracellular matrix in healthy or transformed tissues exhibit more elastic-like or friction-like characteristics over long time scales. Notice that, while our model assumes that the mechanical interactions with the extracellular environment are elastic in nature, it can, in effect, account for friction-like interactions with the environment. This is supported by the fact that cell-matrix elastic interactions are effectively equivalent to friction-like cell-matrix interactions under conditions of high dissociation rates of cell-matrix adhesion bonds.^153,154^ Therefore, this bleb-based friction-based migration regime can be achieved by cells embedded in extracellular matrix within *in vitro* or *in vivo* environments and falls under the bleb-adhesion-based hybrid framework we outline here.

Overall, our work provides a new physical perspective of the possible mechanisms underlying the migration of blebby cells such as T cells and some cancer cells. The developed model not only provides strategies to enhance exploratory bleb-based T cell swimming but also suggests that pushing cells into a hybrid bleb- and adhesion-based mode of migration is a promising engineering strategy to increase T cell migration in tumors. Moreover, our findings suggest that antifibrotic therapies, targeting fibrotic tumor environments, can have contrasting effects on T cell migration, either slowing or enhancing movement depending on the specific treatment, emphasizing the importance of controlled therapeutic interventions. While dense, small-pore matrices may hinder T cell infiltration, intermediate matrix densities can provide the necessary adhesion sites for T cells to grip onto the environment and migrate more effectively.

Additional studies are needed to investigate to what extent this hybrid mode of migration can be adopted by T cells *in vivo*, which presents an exciting avenue for prospective theoretical and experimental research. To achieve this, advanced imaging, cell engineering, and quantitative analysis combined with biophysical modeling will be indispensable in uncovering cell-specific and context-specific migration mechanisms. Ultimately, a faithful model can be used to define optimal states for maximal T cell migration and maximally effective immune cell therapies and thereby guide the development of genetically engineered T cells optimized to migrate through mechanically challenging tumor microenvironments.

### Limitations of the study

Our experimental approach has several limitations. The microgel system recapitulates some, but not all, aspects of the *in vivo* microenvironment. It provides defined interstitial spaces and collagen I-bioconjugated surfaces that support cell adhesion and 3D migration. These interstitial spaces and extracellular matrix composition can be tuned independently in this platform to dissect different modes of T cell migration, but we did not systematically vary these parameters here. In addition, although chemotaxis and durotaxis were not explicitly controlled or quantified in this study, both processes are complex and may influence T cell migratory behavior in meaningful ways. Finally, all experiments were conducted exclusively *in vitro* to enable long-term, continuous *in situ* single-cell tracking, which is not yet feasible with current intravital imaging technologies. We recognize the importance of *in vivo* microenvironmental heterogeneity, and future studies will extend these findings to more physiologically relevant settings.

## RESOURCE AVAILABILITY

### Lead contact

Requests for further information and resources should be directed to and will be fulfilled by the lead contact, Roberto Alonso-Matilla (alons075@umn.edu).

### Materials availability

This study did not generate new, unique reagents.

### Data and code availability

- Data: cell migration data and data analysis codes have been deposited at Zenodo and are publicly available as of the date of publication at https://doi.org/10.5281/zenodo.17676576.^155^
- Code: the codes used for the T cell migration biophysical model have been deposited at Zenodo and are publicly available as of the date of publication at https://doi.org/10.5281/zenodo.17602964.^85^
- All other items: any additional information required to reanalyze the data reported in this paper is available from the lead contact upon request.

## STAR★METHODS

### EXPERIMENTAL MODEL AND STUDY PARTICIPANT DETAILS

#### T cell maintenance

Purified human CD8^+^ T cells (iQ Biosciences, Alameda, CA) were grown in RPMI-1640 supplemented with 10% fetal bovine serum and 2 mM L-glutamine. Recombinant human IL-2 (BioLegend. Cat#589104, San Diego, CA) at the concentration of 100 ng/mL was added to the media for the maintenance of T cells. For activation, T cells were seeded at 3 × 10^6^ cells/mL in 6-well cell-culture treated plates in media containing soluble human anti-CD3 (1 μg/mL) and anti-CD28 (5 μg/mL) antibodies (BioLegend, San Diego, CA) for 48 h.

#### Long-term microscopy assay

Prior to the experiment, T cells were harvested and labeled with CellTracker^™^ Green CMFDA according to the manufacturer’s instructions. An equal number of labeled cells were suspended in 2 groups, collagen-coated and inert microgels, and then seeded into a 96-well plate that has a type #0 coverslip for optimal imaging. Imaging time-lapses were acquired as z-stacks (4um step size at 1024×1024) at 3-minute intervals using a Nikon AXR confocal microscope (Nikon Instruments, Inc USA). The ultra-high sensitive Nikon Spatial Array detector (NSPARC) and high-speed resonant scanning mode were also employed to optimize spatial and temporal resolutions while maintaining low cellular phototoxicity. Cells were maintained at 37°C and 5% CO_2_ in a stage-top incubation chamber (Aurita Biosciences), and timelapse images were acquired with a 10X Plan APO Lambda D air objective (NA 0.45).

### METHOD DETAILS

#### Preparation of collagen-coated and inert poly(acrylamide-co-acrylic acid) microgels

The bioconjugated polyacrylamide microgels were synthesized as previously described.^105,106^ In brief, collagen-conjugated and inert (unconjugated) poly(acrylamide-co-acrylic acid) microgels were fabricated through free radical polymerization using acrylamide monomer, N,N′-methylenebisacrylamide crosslinker (bis-acrylamide, Sigma-Aldrich, Cat# 1003520262, St. Louis, MO, USA), pure acrylic acid (Thermo Scientific, Cat# L04280.36, Waltham, MA, USA), and Acryloxyethyl thiocarbamoyl rhodamine B (Sigma-Aldrich, Cat# 908665, St. Louis, MO, USA). Ammonium persulfate (APS, Sigma-Aldrich, Cat# 248614–100, St. Louis, MO, USA) and TEMED (Fisher Scientific, Cat# B9150–100, Waltham, MA, USA) were added as initiators. The polymerized hydrogel was then mechanically ruptured to create microgel particles. Carboxylic acid groups were activated with EDC (Gibco, Cat# BC25–25, Thermo Fisher Scientific, Waltham, MA, USA) and NHS (Alfa Aesar, Cat# A10312.22, Haverhill, MA, USA) in MES buffer (pH 5.5, Fisher Scientific, Cat# BP300–100, Waltham, MA, USA). Activated microparticles were conjugated with type I bovine collagen (Advanced Biomatrix, Nutragen, Cat# 5010–50, San Diego, CA, USA) at neutral pH. Unreacted sites were quenched with ethanolamine (Sigma-Aldrich, Cat# E6133, St. Louis, MO, USA). Microparticles were thoroughly washed with PBS (1X, Corning, Cat# 21–040-CV, Corning, NY, USA) before use.

#### Datasets and codes

The T cell migration model codes are archived on Zenodo and can be accessed at https://doi.org/10.5281/zenodo.17602964.^85^ All cell migration datasets and corresponding analysis scripts are also available on Zenodo at https://doi.org/10.5281/zenodo.17676576.^155^

### QUANTIFICATION AND STATISTICAL ANALYSIS

A maximum intensity projection was created from z-stacks for each sample. The inherent non-isotropic motion/drift was compensated by optical flow to ensure that the detected motion was referenced to the LLS microgels. The cells were segmented with the Cellpose algorithm.^156^ The centroids of single-segmented cells were tracked with an Image J Linear Assignment Problem (LAP) tracker algorithm. The individual mean squared displacements (iMSD) and mean speeds for each track were calculated, with short detections and those below-the-noise floor filtered out. The histogram of the mean speed of the tracks was plotted, labelling the two distinct populations revealed by the FPT histogram.

## Supplementary Material

1

2

3

4

5

6

7

Supplemental information can be found online at https://doi.org/10.1016/j.celrep.2025.116824.

## Figures and Tables

**KEY RESOURCES TABLE:** 

REAGENT or RESOURCE	SOURCE	IDENTIFIER

Antibodies		

Anti-human CD3	Biolegend	300302
Anti-human CD28	Biolegend	377803

Biological samples		

Purified Human CD8 T Cells	iQ Biosciences	IQB-HU1-CD8T10

Chemicals, peptides, and recombinant proteins		

Recombinant human IL-2	Biolegend	589104
Gibco^™^ L-Glutamine (200 mM)	ThermoFisher Scientific	25030081
HyClone^™^ Characterized Fetal Bovine Serum (FBS)	Cytiva	SH30071.03
Gibco RPMI 1640 medium	ThermoFisher Scientific	21870076
Acrylamide monomer	Sigma-Aldrich	L15075.36
N,N′ -Methylenebisacrylamide (Bisacrylamide)	Sigma-Aldrich	1003520262
Acrylic acid (pure)	Thermo Scientific	L04280.36
Acryloxyethyl thiocarbamoyl rhodamine B	Sigma-Aldrich	908665
Ammonium persulfate (APS)	Sigma-Aldrich	248614-100
TEMED	Fisher Scientific	B9150-100
EDC (1-Ethyl-3-(3-dimethylaminopropyl) carbodiimide)	Gibco, Thermo Fisher Scientific	BC25-25
NHS (N-hydroxysuccinimide)	Alfa Aesar	A10312.22
Type I Bovine Collagen, Nutragen	Advanced Biomatrix	5010-50
Ethanolamine	Sigma-Aldrich	E6133
MES Buffer (pH 5.5)	Fisher Scientific	BP300-100
PBS, 1X	Corning	21-040-CV

Deposited data		

T Cell migration trajectories	10.5281/zenodo.17676576 ^155^	17676576

Experimental models: Cell lines		

Purified Human CD8 T Cells	iQ Biosciences	IQB-HU1-CD8T10

Software and algorithms		

CellPose	https://github.com/MouseLand/cellpose/tree/v3.1.0	3.1.0
Skimage	https://scikit-image.org/docs/stable/release_notes/release_0.22.html	0.22.0
Fiji (ImageJ)	https://github.com/fiji/fiji/tree/fiji-2.16.0	2.16.0
Fortran90/MATLAB	https://doi.org/10.5281/zenodo.17602964 ^85^	17602964

## Highlights

- Hybrid bleb-adhesion-based mode maximizes T cell migration speed
- Adhesion-independent bleb-based swimming is inefficient
- Optimal adhesion strength is required for high cell motility
- Experiments in collagen and inert gels confirm faster migration with modest adhesion
